# Address in Medicine

**Published:** 1903-08-08

**Authors:** 


### ADDRESS IN MEDICINE.

Dr. Frederick T. Roberts gave the address in medicine, having for his subject "Infective and Infectious Diseases." According to our present-day doctrines every infective disease is the result of the direct action on the body of one or more living pathogenic micro-organisms or bacteria, and in many instances it has been shown positively that a specific organism is the cause of a particular disease, and of no other. It must be clearly understood that according to this definition the term "infective" is not synonymous with "infectious," which implies that a complaint is capable of being communicated, either by direct inoculation or contagion, or indirectly in various ways, from person to person, or from some other animal to man. In short, an infectious disease must be infective; but an infective complaint is not necessarily infectious.

The theory of *contagium vivum* is of very ancient date, but the positive demonstration of the nature of the so-called virus was only started and has been progressively accomplished well within the last half-century, while the investigations which are still going on in all directions are adding daily to our knowledge and to the practical results therefrom. These investigations, however, have shown that there are still not a few difficult questions to be solved, and we must be on our guard against too hasty conclusions or generalisations on insufficient data. It is a remarkable fact that in the case of some of the most prominent infectious diseases with which we have long been familiar no definite or specific organism has thus far been recognised; at the same time we may be certain that such an organism does exist, and this fact may be accepted as the basis of a sound working hypothesis.

It is impossible not to be struck with the additions which have been made during the last half-century to this group of infective and infectious diseases, and with the way in which others, though previously known, have come so much more prominently to the front. The multiplication of these complaints has been partly due to the differentiation of diseases which were formerly regarded as identical. It is, however, to the revelations of bacteriology that these additions are mainly due, and which have brought all inflammatory, suppurative, and septic conditions within the infective group.

With regard to the practical etiology of these diseases, micro-organisms have been found and demonstrated in the blood, secretions, excreta, urine as well as fæces, in the cutaneous structures or shed epithelium, possibly in the expired air, and in specific lesions, morbid products, and discharges from the body of various kinds. Hence it is easy to understand how and why most of the diseases with which these organisms are associated are likely te be transmitted from individual to individual; how the infected materials may contaminate fomites, and be thus conveyed far and wide; and how they may be "airborne," being carried about by atmospheric currents or winds, and afterwards either inhaled or swallowed by persons, it may be, far removed from the original source of infection, remote epidemics being thus not uncommonly originated. The communicability of certain grave diseases by the contamination of drinking water is an established fact, the importance and far-reaching consequences of which it is impossible to estimate. One of the most striking additions in modern times to our knowledge regarding the conveyance of infection is in relation to food. In this connection milk stands out prominently. Oysters and other shell fish, including mussels, cockles, and periwinkles, are no doubt agents in originating enteric fever.

The rôle which different animals play in the transmission of infectious diseases to man is another aspect of the subject upon which modern observations and researches have thrown remarkable light. The danger of infection being carried directly by domestic animals, such as birds, cats, and dogs, from person to person, or perhaps because they themselves are suffering from certain diseases, is not yet appreciated as it ought to be. The part played by rats and mice in relation to plague is now familiar to all. Flies, cockroaches, and the smaller personal vermin may, no doubt, be instrumental in conveying infection in certain cases, and flies may taint food. But the most remarkable and important revelations which modern investigators have given as to the transmission of infection by animals are those relating to mosquitos. The discovery and demonstration of the connection between mosquitos and malaria has already worked incalculable good, and promises in the future to revolutionise the conditions of life in many parts of the world.

The question of the relation of infection to soil, place, and particular houses or groups of houses is very important, but is one which needs to be worked out more fully before any positive conclusions can be arrived at with regard to individual diseases.

Another important aspect of infection, from a practical point of view, is the fact that we are every one of us always carrying about in various parts of our bodies microbes, which, while habitually innocuous, may, under favouring conditions, so far as they are concerned, become extremely virulent, to our personal undoing. With regard to the channels of entrance of microbes into the system, the mouth, throat, and neighbouring parts come into unpleasant prominence, especially the tonsils, in connection with scarlatina, rheumatic fever, tuberculosis, actinomycosis and other diseases.

The remainder of Dr. Roberts' address included a consideration of the Morbid Anatomy and Histology, Clinical History and Phenomena and Diagnosis and Treatment and Prevention of the Infective Diseases.

